# Addressing financial and health-related social needs among patients with cancer: Study protocol for CAN-ASSIST pilot clinical trial

**DOI:** 10.1016/j.conctc.2026.101637

**Published:** 2026-04-08

**Authors:** Maedeh Sharifian, Victoria Nguyen, Aarushi Madan, Omar Gutierrez, Mahnur Bharucha, Jeremy Harris, Michael A. Hoyt, Joel Milam, Wen-Pin Chen, Argyrios Ziogas, Gelareh Sadigh

**Affiliations:** a Department of Radiological Sciences, University of California Irvine, CA, USA; b Department of Radiation Oncology, University of California Irvine, CA, USA; c Department of Population Health and Disease Prevention, University of California Irvine, Irvine, CA, USA; d Department of Epidemiology and Biostatistics, University of California Irvine, Irvine, CA, USA; e Chao Family Comprehensive Cancer Center, University of California Irvine, Irvine, CA, USA; f Department of Medicine, University of California Irvine, Irvine, CA, USA

**Keywords:** Cancer, Financial hardship, Health-related social needs, Financial education, Financial navigation, Cost communication

## Abstract

**Background:**

Financial hardship and health-related social needs (HRSNs) are prevalent among cancer patients and contribute to disparities in outcomes. Addressing these issues can reduce harm, yet routine screening and intervention remains underutilized. Navigator-led interventions such as combining out-of-pocket cost (OOPC) communication and financial navigation (CostCOM) have shown promise, but face implementation barriers due to navigator shortages. It is unclear whether financial education and resource access alone (FinEd), without navigator support, can meet patients’ needs.

**Method:**

We propose a three-arm pilot randomized controlled trial comparing FinEd, enhanced usual care (EUC) and CostCOM in 90 newly diagnosed cancer patients undergoing systemic or radiation therapy who screen positive for financial hardship or HRSNs. FinEd includes (1) a list of local/national resources for financial and HRSNs support, and (2) educational materials on health insurance, delivered via mail and a one-time phone/video session with a study coordinator. CostCOM includes (1) systemic therapy OOPC estimates, (2) financial navigation to identify assistance programs, and (3) financial counseling, delivered over two phone/video sessions by a financial navigator. EUC includes study-specific identification of financial hardship and HRSNs. Our goals are to investigate preliminary efficacy of the three arms within six months post-randomization on cost-related care non-adherence (primary outcome), treatment completion, missed appointments, financial worry, material hardship, insurance literacy, quality of life, and sleep quality. We will evaluate patient experience with FinEd using qualitative interviews.

**Conclusion:**

This study will support feasibility for a larger trial, and provide initial efficacy estimates comparing FinEd vs. EUC vs. CostCOM in improving cancer outcomes.

**Trial registration:**

Clinicaltrials.gov NCT06430840; registered 5/24/2024; https://clinicaltrials.gov/study/NCT06430840?cond=NCT06430840&rank=1.

## 1 Introduction

Financial hardship and health-related social needs (HRSN) are significant health equity issues affecting patients in the United States. Approximately 42-56% of cancer survivors report financial hardship and 15-60% report HRSNs [[1], [2], [3], [4], [5], [6], [7]]. Financial hardship is characterized by three domains: cost-related care nonadherence, material hardship (e.g., medical debt), and financial worry [1]. HRSNs are characterized by insecurity about food, housing, transportation, and utilities [8,9]. Both financial hardship and HRSNs contribute to disparities in care access, cancer outcomes, and overall survival, as well as quality of life (QOL) and sleep disturbance [6,8,15]

**Fig. 1:**
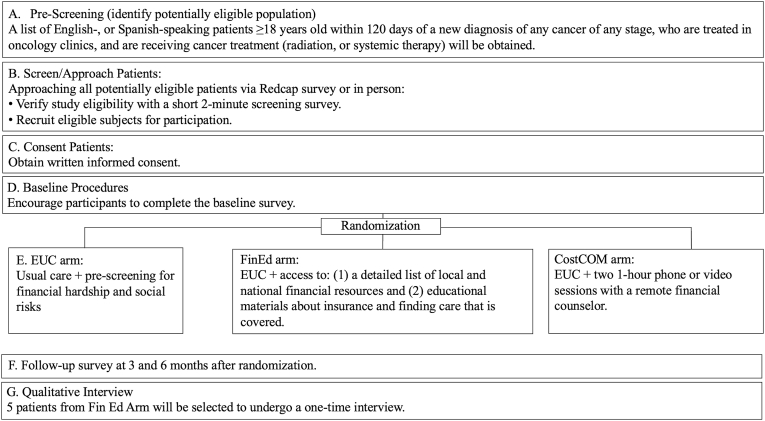
Study schema.

Routine screening for financial hardship and HRSNs in clinical oncology practices is limited by staffing constraints and absence of standardized screening tools [10]. While several screening tools have demonstrated feasibility and acceptability [[16], [17], [18]], and over 70% of community oncology practices report available services to screen for and respond to financial hardship and HRSNs [19], screening remains uncommon as availability of the service does not equate coverage of all patients at risk. Notably only 16.6% of cancer patients were actually screened in a retrospective analysis of a single comprehensive cancer center [10]. This gap highlights the need for approaches that reduce staff burden while addressing patient needs.

Beyond screening, responding to identified needs is also limited by staffing and lack of referral systems [18]. Interventions such as financial navigation, financial education, and cost communication have been proposed [[20], [21], [22]]. While financial navigation improved financial worry among high-risk patients [19], its implementation is constrained by the need for trained navigators. As financial hardship is associated with low financial health literacy [22] and financial self-efficacy [23], financial education may also be effective. However, prior financial education studies have also relied on delivery by navigators [23,24]. It remains unclear whether navigator-free approaches—such as self-directed educational materials and financial resources—are more effective than usual care, or how they compare to active financial navigation in improving financial hardship and quality of life. Further, financial hardship has been associated with poor sleep quality, including increased insomnia severity and sleep disturbances among cancer patients [15]. Given that financial stress is a recognized psychosocial stressor capable of activating physiological pathways that interfere with sleep, interventions targeting financial hardship may plausibly improve sleep outcomes as a secondary benefit. However, the extent to which financial hardship-focused interventions may improve sleep quality among cancer patients remains largely unexplored. Therefore, sleep quality was included as an outcome in the present pilot study to assess whether reductions in financial hardship are accompanied by improvements in this dimension of patient well-being.

The CAN-ASSIST trial is a three-arm pilot randomized controlled trial testing the preliminary efficacy of navigator-free financial education and resources (FinEd) vs. enhanced usual care (EUC) vs. out-of-pocket cost communication and financial navigation (CostCOM) on domains of financial hardship (care non-adherence, material hardship, and financial worry), financial health literacy, quality of life, and sleep quality among cancer patients on systemic treatment who screen positive for financial hardship and HRSNs [15].

## 2 Objectives

We will investigate the preliminary efficacy of FinEd vs. EUC vs. CostCOM within six months after randomization on patient-reported cost-related cancer care non-adherence (primary outcome), electronic medical record (EMR)-abstracted treatment completion, missed appointments, patient-reported financial worry, material financial hardship, health insurance literacy, QOL, and sleep quality as well as objectively measured sleep quality (secondary outcomes). We will further describe FinEd arm patient experience with the FinEd intervention using mixed methods (implementation outcome).

## 3 Materials and methods

### 3.1 Trial design

CAN-ASSIST is a pilot, parallel, 3-arm randomized controlled trial with randomization at patient-level (See Fig. 1). There will be no blinding of participants or study coordinators. The study has been approved by the Institutional Review Board (IRB), and this protocol is reported in accordance with the Standard Protocol Items: Recommendations for Interventional Trials (SPIRIT) guidance [25].

### 3.2 Study site

The study takes place in the six academic and community oncology clinics associated with a single comprehensive cancer center in Southern California. The catchment area of this cancer center includes a diverse population with 43% White, 22% Asian, 2% African American, 14% multi-racial, and 19% other races. Furthermore, 34% of the county's population are Hispanic, 30% are immigrants, and 43% are not a US citizen. A total of 45.2% speak languages other than English at home, with 70% speaking English or Spanish [26].

### 3.3 Eligibility criteria

Patients with the following characteristics are eligible for the study: 1) Age of 18 years or older, 2) being able to answer surveys and interact with the study team in English or Spanish, 3) a new cancer diagnosis of any type or stage by histology in the last 120 days, 4) having started systemic cancer treatment or radiation therapy, 5) being treated in our cancer center oncology clinics, 6) screen positive for financial hardship or HRSNs (see Section 3.4). The 120-day window was selected because prior research shows patients prefer early financial screening [27]. Financial hardship peaks in the first few months after early-stage cancer diagnosis and improves thereafter, [28,29], whereas it worsens over time in metastatic cancer [30], thereby justifying the use of 120 days since diagnosis as an inclusion criterion.

The exclusion criteria included Eastern Cooperative Oncology group (ECOG) performance status more than 2, participation in other therapeutic clinical trials that cover the cost of treatment, prior history of cancer diagnosis or treatment in the last 24 month except for in situ cancers, non-melanoma skin cancers, and breast cancers diagnosed more than 24 months ago who were only receiving hormonal treatment in the last 24 months.

### 3.4 Pre-screening and outreach

Potentially eligible patients will be identified from oncology clinic rosters using a combination of weekly automatic inquiry of electronic medical records (EMR) using institutional IT data warehouse, manual review of clinic schedules, and collaboration with oncology care teams engaged in the study. Patients will be approached both in person by bilingual research coordinators during oncology clinic visits and remotely through email outreach via REDCap.

Interested patients will receive a study information sheet and be asked to complete a brief, 2-min screening survey. This survey will collect basic demographic information (age, gender, race, ethnicity, insurance status) and include five questions to screen for financial hardship and HRSNs. These questions include Item #12 of the Comprehensive Score for Financial Toxicity (COST]) “My illness has been a financial hardship to my family and me” [31,32] and four screening questions addressing food, housing, transportation, and utility insecurity, derived from the Centers for Medicare & Medicaid Services Accountable Health Communities (AHC) Health-Related Social Needs Screening Tool [33]. Patients who endorse any of the four HRSN-related items or financial hardship will be considered eligible for study participation.

To ensure accessibility, the survey will be available in both English and Spanish and can be completed through various convenient methods: in-clinic using paper or electronic forms, online via REDCap email, or by phone with assistance from a bilingual study coordinator.

### 3.5 Baseline survey

Eligible patients will receive a study overview by a study coordinator and provide a signed, dated informed consent on paper (in person or via mail) or electronically (via REDCap). Patients will then be asked to complete a 30-min baseline survey, available in English or Spanish, and in paper or electronic format based on their preference.

The survey includes validated measures to assess patient-reported receipt of cancer supportive services as part of usual care (i.e., cost communication, financial counseling, financial assistance), cost-related care non-adherence [34], material hardship [34], financial worry [31,32], QOL [35], health insurance literacy [36], subjective sleep quality [37], as well as questions about patients’ demographics, insurance, single item health literacy [38], zip code (to assess neighborhood socioeconomic status), and presence of other comorbidities. Participants who complete the baseline survey will receive a $20 gift card.

### 3.6 Randomization

Consented patients who complete the baseline survey will be randomized with a 1:1:1 ratio in a block size of 3 into one of the three study arms generated using PASS 2023 [39]. Randomization will be stratified by cancer stage (1:1:1 non-metastatic vs. metastatic). Metastatic stage is defined by the presence of distant metastasis based on pathological, clinical, or lab findings, or by receipt of non-curative intent treatment. For hematologic malignancies, acute leukemias and stage IV lymphoma will be categorized under metastatic staging.

### 3.7 Study arms

Patients in all arms will receive the usual care, which includes meeting with an institutional social worker upon provider referral due to patient-reported financial and HRSNs concerns during their oncology visits. For all patients, receipt of any institutional financial counseling or navigation will be extracted from EMR by the research coordinators at baseline, 3-, and 6 months after randomization.

#### 3.7.1 Enhanced usual care (EUC)

Usual care will be enhanced by study-specific identification of patients with financial hardship and HRSNs facilitated by the study team. Patient's report of HRSNs or financial hardship will be documented in the study database and is not shared with the clinical team or integrated with the EMR. A prior study has shown screening for financial hardship ***alone*** may reduce the development or worsening of financial hardship [40].

#### 3.7.2 FinEd

Patients will receive FinEd intervention within 4 weeks after enrollment. The intervention consists of mailing patients a packet including: (1) a comprehensive list of local resources that can help with food insecurity, transportation or living expenses; (2) contact information for national non-profit financial navigation organizations such as Patient Advocate Foundation where patients can self-refer. PAF provides case management and financial aid assistance to individuals facing chronic, life-threatening, and debilitating illnesses. [41], and (3) financial education materials in the format of brochures and videos with QR codes on topics such as understanding health insurance costs, how to read an explanation of benefits and medical bills, how to find care that is covered by insurance, how to use price estimator tool (brochure only), choosing a health plan (brochure only). The videos are developed by our research team using content from publicly available materials and input from a team of patients, patient educators, and community advisors working with our cancer center community outreach engagement team. After delivery of the intervention materials, a bilingual study coordinator will set up a one-time phone or zoom meeting with the participants to give an overview of resources shared, and answer patients’ questions. The study coordinator will complete a study-specific training under supervisor of a senior researcher on how to discuss the resources to ensure fidelity in delivery of intervention.

#### 3.7.3 CostCOM

Patients will receive the CostCOM intervention, which is informed by our prior work [22,42] within 4 weeks of enrollment. CostCOM consists of two interactive one-on-one sessions that last up to 1 h in duration. Sessions take place by phone or video at enrollment and 3 months post-randomization with a remote centralized financial navigator using a commercially available price estimator tool and financial navigation platform (TailorMed Inc, New York, NY). TailorMed is a healthcare technology platform that generates OOPC estimates for systemic therapy using patients’ insurance information, and identifies financial assistance programs for which a patient may be eligible for. During these sessions, patients will receive (1) OOPC communication, a review of insurance benefits and education of the patient-specific OOPC estimate for anticipated systemic treatment regimen obtained using the price estimator tool; (2) Financial navigation: real-time professional guidance to identify financial assistance programs (e.g., co-pay, living expenses) that alleviate costs of care using the financial navigation platform as well as discussion of strategies to improve insurance coverage; and (3) Financial counseling to address the range of patients' financial concerns and enroll patients in financial assistance programs. At the end of each session, the financial navigator will generate a report with data on information discussed, patient enrollments in assistance programs, and the monetary amount of assistance. This data will be shared with the patient.

### 3.8 Follow-up surveys

All patients will be surveyed at 3- and 6-months post-randomization to assess patient-reported receipt of cancer supportive services as part of usual care, cost-related cancer care nonadherence [34], material hardship [34], financial worry [31,32], QOL [35], health insurance literacy [43], subjective sleep quality [37], and patients’ experience with the CostCOM and FinEd interventions. Surveys will be distributed electronically via REDCap or by mail, with a pre-stamped, pre-addressed return envelope, according to their preferences. Non-responders of the survey will be approached to complete the survey via phone or in the clinic. Patients will be sent $30 and $40 gift cards after completion of the 3- and 6-month surveys, respectively.

### 3.9 Sleep study procedures

Participants may opt to take part in an optional sleep monitoring sub study, which involves objective assessment of sleep quality using wrist actigraphy (Ambulatory monitoring inc., Ardsley, NY)—a noninvasive, ecologically valid method widely used to measure sleep-wake patterns [[44], [45], [46]]. Wrist actigraphy has been validated against electroencephalogram (EEG) measures and shows strong agreement with polysomnography, with correlations exceeding 90% in adults [[44], [45], [46]].

Interested participants will be asked to wear an actigraph on their non-dominant wrist continuously (24 h/day) for 7 consecutive days at three time points: within 3 weeks of enrollment, and at 3- and 6-months post-randomization, in accordance with standard recommendations [44]. Participants will begin actigraphy monitoring before receiving study intervention at any time. To support accurate data interpretation, participants will also complete a daily actigraphy log during each 7-day monitoring period. The log will capture the time the participant got into bed, the time they attempted to fall asleep, the time of final awakening in the morning, the time they got out of bed for the day, the timing and duration of any daytime naps, periods when the actigraph was removed or reattached, and any unusual events that could affect sleep or wake patterns (e.g., illness, travel, or prolonged sedentary activity).

The actigraphy kit—including the device, an instructional guide, a cover letter, and a pre-stamped, pre-addressed return envelope—will be mailed to participants by the study team.

### 3.10 Qualitative interviews

We will conduct one-on-one qualitative interviews with 5 participants from the FinEd arm who consented to these interviews at the enrollment. This sample size was selected based on availability of funding and resources to refine the intervention prior to larger-scale evaluation. Selection of participants will be purposeful based on participants' responses to follow-up surveys indicating use of FinEd intervention materials. These interviews will be conducted via phone or video to assess participants' experiences and satisfaction with the FinEd intervention. Each interview is expected to last approximately 30–45 min. Participants with a completed interview will receive a $40 gift card. Given our team have previously conducted qualitative interviews with patients receiving the CostCOM intervention [22], we will not interview any patients from this arm.

### 3.11 Patient assessments

#### 3.11.1 Primary efficacy outcomes

The primary outcome is patient-reported cost-related cancer care nonadherence within 6 months post-randomization. Cost-related cancer care nonadherence is defined as a positive response to any of the following: (1) delay, (2) forego, (3) stop, or (4) change in cancer prescribed medication due to cost, (5) refuse recommended cancer tests, or (6) skip cancer office visits due to cost, adapted from Medical Expenditure Panel Survey [34]. At each of the 3-, and 6-month follow-ups, patients will be asked about the presence of nonadherence (yes/no) since the last follow-up. Cost-related cancer care nonadherence at 6 months will be calculated as a report of nonadherence at any of the 3 and 6-month follow-ups (yes/no). We will also evaluate the presence of cost-related cancer care non-adherence at each time point separately.

#### 3.11.2 Secondary efficacy outcomes

Secondary outcomes and their definitions are shown in Table 1 and include treatment completion [47] (yes/no), rate of missed appointments [47], and material financial hardship [34] within 6 months post randomization, as well as changes in patient-reported financial worry [31,32], health insurance literacy [43], QOL [35], subjective sleep quality [37] between baseline and 3- and 6-months. A sleep quality parameters will be calculated using the Action-W program and include: sleep duration (total nighttime sleep minutes), sleep efficiency (ratio of nighttime sleep duration to total sleep period), sleep latency (minutes to first sleep epoch), and sleep fragmentation (ratio of number of nighttime awakenings to nighttime sleep duration ∗ 100). The index of sleep fragmentation considers movements of varying intensity [43].

**Table 1:** Preliminary Efficacy Outcome Measures and co-variates.

Outcome	Definition	Source
Cost-related cancer care nonadherence *(****Primary)***	Any incidence during the 6 months (measured at 3, and 6 months) when a patient self-reports a positive response to any of: (1) delay, (2) forego, (3) stop, or (4) change in cancer prescribed medication due to cost, (5) refuse recommended cancer tests, or (6) skip cancer office visits due to cost (adapted from Medical Expenditure Panel Survey) [34]. The outcome will be binary (yes/no).	Patient surveys at 3& 6 months. Outcome will also be measured at baseline to control baseline data.
Treatment completion	Receipt of the prescribed cycle of chemotherapy and/or radiation therapy due within the 6-month study period, determined by EMR abstraction (Yes/No) [47]. Patients with treatment interruptions/stoppages because of toxicities, other medical reasons, facility factors, or death will be considered as having completed treatment if they complete all recommended treatment within the 6-month interval. Treatment interruptions/stoppages without medical reasons will be considered incomplete treatment.	EMR
Missed appointment	Proportion of outpatient medical or radiation oncology treatment appointments (including infusion) or office visits (including tele medicine) that is marked as cancellation or no shows and are provider-initiated or are not due to scheduling errors [47]	EMR
Material financial hardship	Any incidence during the 6 months (measured at 3, and 6, months) when a patient self-reports a positive response to any of the following: (1) home sale, refinance or move to affordable rental, (2) loans, (3) reaching credit limits, and (4) bankruptcy) because of your cancer care, or its treatment (adapted from Medical Expenditure Panel Survey) [34]. The outcome will be binary (yes/no).	Patient surveys at 3& 6 months. Outcome will also be measured at baseline to control baseline data.
Financial worry	11-item Comprehensive Score for Financial Toxicity (COST) [31,32]. (Score 0-44; higher score = lower financial worry)	Patient surveys at 3& 6 months. Outcome will also be measured at baseline to control baseline data.
Health Insurance literacy	21-item Health Insurance Literacy Measure [43]. (score 21-84); higher score = higher health insurance literacy)	
QOL	10-item Patient-Reported Outcomes Measurement Information System (PROMIS)-10 version 1.2 [35]. (Score 0-100; higher score = higher quality of life)	
Patient-reported sleep quality	Insomnia Severity Index (ISI). 7-item that evaluates perceived severity of insomnia symptoms [37]. (score 0-28). Score of 10 = clinically significant insomnia	
Total sleep time	The total duration of sleep episodes, calculated from the time the individual falls asleep to the time they wake up [53]	Sleep actigraph
Sleep efficiency	The percentage of time spent asleep while in bed, calculated as (Total Sleep Time/Time in Bed) ∗ 100 [53]	Sleep actigraph
Nighttime awakening	The number of times the individual wakes up during the night [53]	Sleep actigraph
Sleep latency	The time it takes to fall asleep after getting into bed, measured as the time difference between bedtime and sleep onset [53]	Sleep actigraph
**Co-Variates**		
Sociodemographic	Age, sex, race, ethnicity, marital status, education, employment status, annual household income, address and zip codes (to calculate area deprivation index [54])	Patient survey & EMR at baseline and updated at 3, and 6 months
Insurance	Insurance type	
Health Literacy	Single item Health Literacy [48] (score 1-5; higher score = higher health literacy)	Survey
Use of supportive services	Receipt of out-of-pocket cost communication, financial counseling or assistance as part of usual care	Survey
Comorbidities	NCI morbidity index [39]	Survey
Cancer variables	Type, stage, treatment	
Medical history	ECOG performance status (0, 1 & 2)	

#### 3.11.3 Co-variates

We will collect variables such as patient sociodemographic and insurance via surveys and EMR data. Surveys will also include questions to assess health literacy [48], use of supportive services, and comorbidities. Cancer type, stage and treatment, and ECOG performance scale at enrollment [49] will be abstracted from EMR.

#### 3.11.4 Implementation outcomes

Implementation outcomes for patients in the FinEd arm include patients' report of use of resources received in FinEd arm, and their perception of comprehensibility, and helpfulness of these resources measured via follow-up surveys. We will further assess patients' experience with FinEd resources through semi-structured one-on-one qualitative interviews. The interview evaluation will be guided by the Reach, Effectiveness, Adoption, Implementation, and Maintenance (RE-AIM) framework [50] to assess the intervention's overall impact and effectiveness prior to larger-scale evaluation. For patients in CostCOM arm, we will assess patients' satisfaction with the financial counseling session using follow-up surveys.

### 3.12 Statistical analysis

#### 3.12.1 Power calculation and sample size

This is a 3-arm pilot randomized controlled trial to gather preliminary data to plan for a large future trial. A sample size of 90 patients (30 patients in each arm) is feasible given the limits of the study timeline and will allow us to characterize key patient variables.

Power analysis was performed to estimate a one-sided 85%, 90% and 95% upper-limit confidence intervals for proportion of patient-reported cost-related cancer care nonadherence at 6 months post-randomization in an experimental arm via the Exact (Clopper-Pearson) formula when the evaluable sample size for a study arm is 30 (Table 2). With an evaluable sample size of 30 in an experimental arm and the anticipated proportion is 0.05, the estimated 95% upper-limit confidence limit is 0.17.

**Table 2:** Estimation of one-sided upper-limit confidence interval for primary outcome via the Clopper-Pearson Exact method when the evaluable sample size of 30 in an experimental arm.

The proportion of patient-reported cost-related cancer care nonadherence at 6 months post-randomization in an experimental arm (P)	One-sided upper limit of a confidence interval via the Clopper-Pearson Exact method		
	85%	90%	95%
0.05	0.1296	0.14613	0.17251
0.1	0.19052	0.2093	0.2386
0.15	0.24806	0.26832	0.29948

SPIRIT 2025 checklist of items to address in a randomized trial protocol^a^			
Section/Topic	No	SPIRIT 2025 checklist item description	Reported on page no.
**Administrative information**			
Title and structured summary	1a	Title stating the trial design, population, and interventions, with identification as a protocol	1
	1b	Structured summary of trial design and methods, including items from the World Health Organization Trial Registration Data Set	NA
Protocol version	2	Version date and identifier	NA
Roles and responsibilities	3a	Names, affiliations, and roles of protocol contributors	1
	3b	Name and contact information for the trial sponsor	16
	3c	Role of trial sponsor and funders in design, conduct, analysis, and reporting of trial; including any authority over these activities	16
	3d	Composition, roles, and responsibilities of the coordinating site, steering committee, endpoint adjudication committee, data management team, and other individuals or groups overseeing the trial, if applicable	NA
**Open science**			
Trial registration	4	Name of trial registry, identifying number (with URL), and date of registration. If not yet registered, name of intended registry	3
Protocol and statistical analysis plan	5	Where the trial protocol and statistical analysis plan can be accessed	NA
Data sharing	6	Where and how the individual de-identified participant data (including data dictionary), statistical code, and any other materials will be accessible	16
Funding and conflicts of interest	7a	Sources of funding and other support (e.g., supply of drugs)	16
	7b	Financial and other conflicts of interest for principal investigators and steering committee members	17
Dissemination policy	8	Plans to communicate trial results to participants, healthcare professionals, the public, and other relevant groups (e.g., reporting in trial registry, plain language summary, publication)	NA
**Introduction**			
Background and rationale	9a	Scientific background and rationale, including summary of relevant studies (published and unpublished) examining benefits and harms for each intervention	4
	9b	Explanation for choice of comparator	4-5
Objectives	10	Specific objectives related to benefits and harms	5
**Methods: Patient and public involvement, trial design**			
Patient and public involvement	11	Details of, or plans for, patient or public involvement in the design, conduct, and reporting of the trial	NA
Trial design	12	Description of trial design including type of trial (e.g., parallel group, crossover), allocation ratio, and framework (e.g., superiority, equivalence, non-inferiority, exploratory)	5
**Methods: Participants, interventions, and outcomes**			
Trial setting	13	Settings (e.g., community, hospital) and locations (e.g., countries, sites) where the trial will be conducted	5
Eligibility criteria	14a	Eligibility criteria for participants	6
	14b	If applicable, eligibility criteria for sites and for individuals who will deliver the interventions (e.g., surgeons, physiotherapists)	NA
Intervention and comparator	15a	Intervention and comparator with sufficient details to allow replication including how, when, and by whom they will be administered. If relevant, where additional materials describing the intervention and comparator (e.g., intervention manual) can be accessed	6-11
	15b	Criteria for discontinuing or modifying allocated intervention/comparator for a trial participant (e.g., drug dose change in response to harms, participant request, or improving/worsening disease)	NA
	15c	Strategies to improve adherence to intervention/comparator protocols, if applicable, and any procedures for monitoring adherence (e.g., drug tablet return, sessions attended)	NA
	15d	Concomitant care that is permitted or prohibited during the trial	8
Outcomes	16	Primary and secondary outcomes, including the specific measurement variable (e.g., systolic blood pressure), analysis metric (e.g., change from baseline, final value, time to event), method of aggregation (e.g., median, proportion), and time point for each outcome	11-12
Harms	17	How harms are defined and will be assessed (e.g., systematically, non-systematically)	NA
Participant timeline	18	Time schedule of enrollment, interventions (including any run-ins and washouts), assessments, and visits for participants. A schematic diagram is highly recommended (see Figure)	6-12
Sample size	19	How sample size was determined, including all assumptions supporting the sample size calculation	13
Recruitment	20	Strategies for achieving adequate participant enrollment to reach target sample size	6
**Methods: Assignment of interventions**			
Randomization:			
Sequence generation	21a	Who will generate the random allocation sequence and the method used	8
	21b	Type of randomization (simple or restricted) and details of any factors for stratification. To reduce predictability of a random sequence, other details of any planned restriction (e.g., blocking) should be provided in a separate document that is unavailable to those who enroll participants or assign interventions	8
Allocation concealment mechanism	22	Mechanism used to implement the random allocation sequence (e.g., central computer/telephone; sequentially numbered, opaque, sealed containers), describing any steps to conceal the sequence until interventions are assigned	8
Implementation	23	Whether the personnel who will enroll and those who will assign participants to the interventions will have access to the random allocation sequence	NA
Blinding	24a	Who will be blinded after assignment to interventions (e.g., participants, care providers, outcome assessors, data analysts)	5
	24b	If blinded, how blinding will be achieved and description of the similarity of interventions	NA
	24c	If blinded, circumstances under which unblinding is permissible, and procedure for revealing a participant's allocated intervention during the trial	NA
**Methods: Data collection, management, and analysis**			
Data collection methods	25a	Plans for assessment and collection of trial data, including any related processes to promote data quality (e.g., duplicate measurements, training of assessors) and a description of trial instruments (e.g., questionnaires, laboratory tests) along with their reliability and validity, if known. Reference to where data collection forms can be accessed, if not in the protocol	7-11
	25b	Plans to promote participant retention and complete follow-up, including list of any outcome data to be collected for participants who discontinue or deviate from intervention protocols	10
Data management	26	Plans for data entry, coding, security, and storage, including any related processes to promote data quality (e.g., double data entry; range checks for data values). Reference to where details of data management procedures can be accessed, if not in the protocol	11-12
Statistical methods	27a	Statistical methods used to compare groups for primary and secondary outcomes, including harms	14
	27b	Definition of who will be included in each analysis (e.g., all randomized participants), and in which group	14
	27c	How missing data will be handled in the analysis	13
	27d	Methods for any additional analyses (e.g., subgroup and sensitivity analyses)	NA
**Methods: Monitoring**			
Data monitoring committee	28a	Composition of data monitoring committee (DMC); summary of its role and reporting structure; statement of whether it is independent from the sponsor and funder; conflicts of interest and reference to where further details about its charter can be found, if not in the protocol. Alternatively, an explanation of why a DMC is not needed	NA
	28b	Explanation of any interim analyses and stopping guidelines, including who will have access to these interim results and make the final decision to terminate the trial	NA
Trial monitoring	29	Frequency and procedures for monitoring trial conduct. If there is no monitoring, give explanation	11-12
**Ethics**			
Research ethics approval	30	Plans for seeking research ethics committee/institutional review board approval	5
Protocol amendments	31	Plans for communicating important protocol modifications to relevant parties	NA
Consent or assent	32a	Who will obtain informed consent or assent from potential trial participants or authorized proxies, and how	6
	32b	Additional consent provisions for collection and use of participant data and biological specimens in ancillary studies, if applicable	NA
Confidentiality	33	How personal information about potential and enrolled participants will be collected, shared, and maintained in order to protect confidentiality before, during, and after the trial	6
Ancillary and post-trial care	34	Provisions, if any, for ancillary and post-trial care, and for compensation to those who suffer harm from trial participation	NA

Citation: Chan A-W, Boutron I, Hopewell S, Moher D, Schulz KF et al. SPIRIT 2025 statement: updated guideline for protocols of randomised trials. BMJ 2025; 389:e081477. https://dx.doi.org/10.1136/bmj-2024-081477.

© 2025 Chan A-W et al. This is an Open Access article distributed under the terms of the Creative Commons Attribution License (https://creativecommons.org/licenses/by/4.0/), which permits unrestricted use, distribution, and reproduction in any medium, provided the original work is properly cited.

a We strongly recommend reading this checklist in conjunction with the SPIRIT 2025 Explanation and Elaboration and the SPIRIT 2025 Expanded Checklist for important clarifications on all the items. We also recommend reading relevant SPIRIT extensions. See www.consort-spirit.org.

#### 3.12.2 Approach to managing missing data

We will evaluate baseline characteristics across study groups to determine if randomization effectively balances covariates. All outcome analyses will follow an intent-to-treat approach. Participant dropout will be closely monitored to assess whether the missing data is informative. Additionally, we will examine how differences in dropout rates between groups may influence the primary and secondary outcomes.

#### 3.12.3 Analysis plan

We will report proposed outcome measures using descriptive measures. A difference and its 95% confidence interval in an outcome endpoint will be estimated via conducting pairwise comparisons between each intervention arm (***CostCOM*** or ***FinEd***) versus ***EUC*** arm. The Exact (Clopper-Pearson) method will be utilized to obtain a 95% confidence interval. Logistic regressions will be used to evaluate the impact of potential co-variates on primary endpoint among study arms. Linear regression analysis will be utilized to model a continuous outcome. For the longitudinal outcomes, we will implement the Generalized Estimating Equations (GEE) approach to adjust for the systematically different observation periods, in which time will be modeled as a fixed effect at each step**.**

#### 3.12.4 Analysis of qualitative interviews

We will apply conventional thematic analysis techniques. All audio-recorded interviews will be transcribed verbatim. An initial codebook will be developed based on key evaluation questions and themes that emerge from open coding of the first few transcripts, followed by iterative team discussions to refine the framework [51]. We will use node reports—collections of coded text segments—to support the identification of sub-themes. Patient-level data will be organized into matrices, incorporating illustrative quotes [52].

## 4 Discussion

Financial hardship and HRSNs are persistent, under-addressed barriers in cancer care that contribute significantly to disparities in treatment adherence, quality of life (QOL), and clinical outcomes [6,[8], [9], [10], [11], [12], [13], [14],^20^]. Despite growing recognition of their impact, scalable and sustainable solutions remain limited. Our protocol seeks to evaluate the feasibility, acceptability, and preliminary efficacy of a navigator-free intervention—FinEd—as a potentially cost-effective and scalable alternative to the previously studied CostCOM intervention, which requires trained financial navigators. We aim to compare FinEd, CostCOM, and EUC among cancer patients who screen positive for financial hardship or HRSNs across a range of patient-reported health outcomes.

This protocol introduces several key innovations that address critical gaps in cancer care related to financial hardship and HRSNs. By focusing on populations at heightened risk for financial hardship and HRSNs, and who are disproportionately affected by health disparities, this study directly targets underserved and vulnerable groups. To our knowledge, this is the first study to compare FinEd, a scalable, navigator-free model that empowers patients through education and self-advocacy with usual care as well as CostCOM, a financial navigation-based intervention. The comparison of these distinct approaches will indicate relative effectiveness and inform future large-scale efforts. Moreover, the centralized delivery of both interventions—CostCOM via remote navigators and FinEd via trained research staff—reduces variability, enhancing methodological rigor. Finally, this study is novel in assessing the impact of financial interventions on sleep quality—a frequently overlooked yet highly burdensome symptom among cancer patients. Up to 79% of cancer patients experience sleep disturbances during treatment, and cancer survivors report sleep problems at rates 11–32% higher than non-cancer controls [15]. Moreover, cancer-related financial hardship has been associated with a 1.58-fold increased risk of long-term, impairing sleep problems [15].

Our findings will yield actionable information to inform and support future interventions to reduce financial hardship and HRSNs and promote care adherence, especially when there is navigator staffing shortage.

## CRediT authorship contribution statement

**Maedeh Sharifian:** Writing – review & editing, Writing – original draft, Methodology, Investigation. **Victoria Nguyen:** Writing – review & editing, Methodology, Investigation. **Aarushi Madan:** Writing – review & editing, Methodology, Investigation. **Omar Gutierrez:** Writing – review & editing, Methodology, Investigation. **Mahnur Bharucha:** Writing – review & editing, Methodology, Investigation. **Jeremy Harris:** Writing – review & editing, Methodology, Investigation, Conceptualization. **Michael A. Hoyt:** Writing – review & editing, Methodology, Investigation, Conceptualization. **Joel Milam:** Writing – review & editing, Methodology, Investigation, Conceptualization. **Wen-Pin Chen:** Writing – review & editing, Methodology. **Argyrios Ziogas:** Writing – review & editing, Methodology. **Gelareh Sadigh:** Writing – review & editing, Writing – original draft, Supervision, Resources, Project administration, Methodology, Investigation, Funding acquisition, Conceptualization.

## Ethics approval and consent to participate

The study has been approved by the University of California Irvine Institutional Review Board (IRB)#4937. All participants complete a consent form and HIPAA authorization form. However, no human subject data is reported in this protocol paper.

## Consent for publication

Not applicable.

## Availability of data and materials

Data sharing is not applicable to this article as no datasets were generated or analyzed during the current study.

## Funding

Research reported in this publication was supported in part by the 10.13039/100000054National Cancer Institute of the 10.13039/100000002National Institutes of Health under award number P30CA062203 and the UC 10.13039/100008476Irvine Chao Family Comprehensive Cancer Center using UCI Anti-Cancer Challenge funds. The authors wish to acknowledge the support of the 10.13039/100009819Chao Family Comprehensive Cancer Center Biostatistical and Biobehavioral Shared Resource, supported by the 10.13039/100000054National Cancer Institute of the 10.13039/100000002National Institutes of Health under award number P30CA062203. The content is solely the responsibility of the authors and does not necessarily represent the official views of the National Institutes of Health.

## Declaration of competing interest

The authors declare the following financial interests/personal relationships which may be considered as potential competing interests:G. Sadigh receives honorarium from the *Journal of the American College of Radiology* in her role as Associate Editor. G Sadigh, M Hoyt, J Milam receive research support from NIH/NCI. Other authors reported no conflicts of interests.

## Figures and Tables

**Table:** 

AHC	Accountable Health Communities
CostCOM	Out-of-pocket cost (OOPC) communication and financial navigation
COST	Comprehensive Score for Financial Toxicity
EMR	Electronic Medical records
EUC	Enhanced usual care
FinEd	Financial education and resource access
HRSN	Health-related social needs
IRB	Institutional Review Board
OOPC	Out-of-pocket cost
PAF	Patient Advocate Foundation
QOL	Quality of life
SPIRIT	Standard Protocol Items: Recommendations for Interventional Trials

## Data Availability

No data was used for the research described in the article.
